# Quantitative Identification of Functional Connectivity Disturbances in Neuropsychiatric Lupus Based on Resting-State fMRI: A Robust Machine Learning Approach

**DOI:** 10.3390/brainsci10110777

**Published:** 2020-10-25

**Authors:** Nicholas John Simos, Stavros I. Dimitriadis, Eleftherios Kavroulakis, Georgios C. Manikis, George Bertsias, Panagiotis Simos, Thomas G. Maris, Efrosini Papadaki

**Affiliations:** 1 Computational Bio-Medicine Laboratory, Institute of Computer Science, Foundation for Research and Technology–Hellas, 70013 Heraklion, Greece; nicholasjohnsimos@gmail.com (N.J.S.); gmanikis@ics.forth.gr (G.C.M.); tmaris@med.uoc.gr (T.G.M.); fpapada@otenet.gr (E.P.); 2 Department of Electrical and Computer Engineering, Technical University of Crete, 73100 Chania, Greece; 3 Integrative Neuroimaging Lab, 55133 Thessaloniki, Greece; stidimitriadis@gmail.com; 4 1st Department of Neurology, G.H. “AHEPA”, School of Medicine, Faculty of Health Sciences, Aristotle University of Thessaloniki (AUTH), 54124 Thessaloniki, Greece; 5 Neuroinformatics Group, Cardiff University Brain Research Imaging Centre (CUBRIC), School of Psychology, College of Biomedical and Life Sciences, Cardiff University, Cardiff CF24 4HQ, UK; 6 Division of Psychological Medicine and Clinical Neurosciences, Neuroscience and Mental Health Research Institute School of Medicine, & MRC Centre for Neuropsychiatric Genetics and Genomics, College of Biomedical and Life Sciences, Cardiff University, Cardiff CF14 4EP, UK; 7 Department of Radiology, Medical School, University of Crete, University Hospital of Heraklion, 71003 Heraklion, Greece; terryka21985@gmail.com; 8 Department of Rheumatology, Clinical Immunology and Allergy, Medical School, University of Crete, University Hospital of Heraklion, 71003 Heraklion, Greece; gbertsias@uoc.gr; 9 Institute of Molecular Biology and Biotechnology, Foundation for Research and Technology–Hellas, 70013 Heraklion, Greece; 10 Department of Psychiatry, Medical School, University of Crete, University Hospital of Heraklion, 71003 Heraklion, Greece

**Keywords:** neuropsychiatric systemic lupus erythematosus, rs-fMRI, graph theory, functional connectivity, surrogate data, machine learning, visuomotor ability, mental flexibility

## Abstract

Neuropsychiatric systemic lupus erythematosus (NPSLE) is an autoimmune entity comprised of heterogenous syndromes affecting both the peripheral and central nervous system. Research on the pathophysiological substrate of NPSLE manifestations, including functional neuroimaging studies, is extremely limited. The present study examined person-specific patterns of whole-brain functional connectivity in NPSLE patients (*n* = 44) and age-matched healthy control participants (*n* = 39). Static functional connectivity graphs were calculated comprised of connection strengths between 90 brain regions. These connections were subsequently filtered through rigorous surrogate analysis, a technique borrowed from physics, novel to neuroimaging. Next, global as well as nodal network metrics were estimated for each individual functional brain network and were input to a robust machine learning algorithm consisting of a random forest feature selection and nested cross-validation strategy. The proposed pipeline is data-driven in its entirety, and several tests were performed in order to ensure model robustness. The best-fitting model utilizing nodal graph metrics for 11 brain regions was associated with 73.5% accuracy (74.5% sensitivity and 73% specificity) in discriminating NPSLE from healthy individuals with adequate statistical power. Closer inspection of graph metric values suggested an increased role within the functional brain network in NSPLE (indicated by higher nodal degree, local efficiency, betweenness centrality, or eigenvalue efficiency) as compared to healthy controls for seven brain regions and a reduced role for four areas. These findings corroborate earlier work regarding hemodynamic disturbances in these brain regions in NPSLE. The validity of the results is further supported by significant associations of certain selected graph metrics with accumulated organ damage incurred by lupus, with visuomotor performance and mental flexibility scores obtained independently from NPSLE patients.

## 1. Introduction

Resting-state functional MRI (rs-fMRI) is a non-invasive functional neuroimaging modality based on the blood-oxygen-level-dependent (BOLD) signal. During data acquisition, subjects are not performing any specific mental or physical task. Thus, rs-fMRI can capture the intrinsic, innate or “default” processes, self-referential thought and introspection that are active even while subjects are at rest. This poses several challenges in experimental design, especially in appropriate analysis methodology. This is different from task-related functional neuroimaging studies, where several proposed methodologies attempt to identify patterns that in some form correspond to or correlate with the task or experiment performed during the examination. In recent years, rs-fMRI has shown great potential as a useful tool in aiding disease diagnosis. Additionally, useful and possibly novel biomarkers can emerge from state-of-the-art rs-fMRI analyses, further enhancing the understanding and interpretation of certain diseases, conditions, pathologies or neural processes.

Systemic lupus erythematosus (SLE) is a chronic, multisystem, autoimmune disease often accompanied by miscellaneous neuropsychiatric manifestations (neuropsychiatric SLE (NPSLE)). Despite systematic attempts to establish universally accepted diagnostic protocols [1], diagnosis of NPSLE as well as attribution of NP manifestations to the pathophysiology of lupus is often challenging. Brain pathology in NPSLE may include small vessel vasculopathy and vasculitis [2], which may in turn be responsible for regional hemodynamic disturbances [3]. Two recent studies using different MRI techniques have documented decreased cerebral blood flow in normal-appearing white matter [4] and cortex [5] in multiple frontal and parietal areas in NPSLE as compared to healthy individuals. Studies utilizing rs-MRI to assess functional connectivity disturbances in NPSLE are scarce, with findings indicating both reduced and increased functional connectivity in NPSLE patients as compared to healthy controls within several a-priori defined networks [6].

Given the vast number of potentially relevant features that can be derived from rs-fMRI examinations, there is growing interest in machine learning (ML) or deep learning (DL) algorithms for feature reduction and patient classification in a wide range of neurological and psychiatric disorders, including multiple sclerosis [7], schizophrenia [8], Alzheimer’s disease [9], attention deficit hyperactivity disorder [10] and autism [11]. Functional connectivity (FC)-based measures were utilized in many such studies as a way of capturing topological neural interconnections and communications of distinct brain areas during rest. FC can be generally considered as a transformation of the initial high-dimensional fMRI data, quantified by simple metrics such as correlation, covariance or mutual information calculated between the recorded time series of different brain regions. Adaptive algorithms utilizing functional neuroimaging data have not been employed thus far on SLE or NPSLE patients, with the exception of a preliminary study utilizing connectivity indices derived from conventional, a-priori defined functional networks [12].

The work presented here builds upon an earlier attempt from our group [12] to tackle the diagnostic problem related to NPSLE. In the previous study, we presented and evaluated a method for the effective classification of NPSLE patients from healthy age-matched participants using an ML classification model. A set of brain networks were selected a-priori based on results of similar studies and the findings of several studies concerning resting-state networks [13]. The obvious limitation of this approach is that it may obscure individual patterns of functional connectivity disturbances in the population of NPSLE patients and assumes that connectivity within each prespecified network is consistently impaired in this disorder. To address this issue, we designed and applied a pipeline that considers and preserves individual differences in the precise outline of functional connectivity patterns. In the present study, we propose a robust automated machine learning (AutoML) approach based on individualized static functional connectivity graphs (sFCG) with the goal of defining objective connectomic biomarkers related to NPSLE. An integral component of the proposed approach entails the use of statistical topologic filtering of individual sFCGs using appropriate surrogate time series.

### 1.1. Deriving Individualized Whole-Brain Network Metrics

#### 1.1.1. Initial Network Specification

To ensure that all cortical and subcortical (telencephalic and diencephalic) brain areas were taken into account in producing a robust model, we computed functional connectivity metrics within an inclusive set of brain areas as specified in a commonly used brain atlas (automated anatomical labeling atlas (AAL)) [14].

#### 1.1.2. Statistical Thresholding of Functional Connections

Given the exceedingly high number of potential features (complementary metrics characterizing each of the 4005 implied functional connections and regions) we designed a rigorous data-driven protocol for feature reduction. The proposed method comprised statistical filtering applied to the complete matrix of connectivity weights between all possible pairs of regions using person-specific statistical thresholds. The first step entails constructing a static functional connectivity graph (FCG), a single adjacency matrix containing the connectivity weights between all possible pairs of regions. The dynamic and somewhat unpredictable nature of two resting-state fMRI regional time courses renders application of a fixed statistical threshold (e.g., Fisher’s z value) untenable. Comparison of observed connectivity values to the distribution of such indices computed from synthetic (surrogate) time series is one option employed extensively on electric and magnetic time series [15,16,17]. Unbiased statistical testing, however, requires that the surrogate sets share the natural variability of underlying dynamic systems so that the connectivity weights computed between surrogate time series closely represent the morphological and spectral characteristics of the original time series. At the same time, surrogate data should not share features of systematic, underlying neuronal activation. Comparing a given “true” connection to those computed between multiple surrogate time series (in the form of a distribution) can reveal whether that connection is significant, i.e., contains useful information about the underlying neuronal system. The data driven graph thresholding technique utilized in this study was implemented using iterated amplitude adjusted Fourier transform surrogate time-series (IAAFT) [18]. IAAFT surrogates were developed by [18] to address the flatness bias problem observed in the power spectra of conventional AAFT time series. The Fourier amplitudes are iteratively replaced, and the distributions rescaled in order to best match the original time series on both amplitude distribution and power spectrum. Alternative, fixed-thresholding schemes (as for instance retaining only connections with the same node degree [19]) were considered as too arbitrary to capture the complex pathophysiology of NPSLE. Previously proposed data-driven methods have produced promising results and were also considered in the preliminary stages of the present work, namely orthogonal minimum spanning trees [20]. The latter is a variant of minimum spanning trees [21], where multiple “independent” trees are created from the graph instead of a single tree containing all of the original graph nodes.

#### 1.1.3. Graph Theory Network Metrics

Computation of a common set of metrics which best characterize each reduced FCG is a crucial step to ensure that a common set of metrics is fed to the ML/DL classification model for every participant. The utilization of person-specific FCG reduction techniques leads to potentially different sparse FCGs for each participant. As a result, the connectivity values (FCG edges) cannot be used as the feature vector at this stage. One proposed solution utilized in several studies [22,23,24] involves graph (or network) metrics. In [24], graph metrics provided promising results when used on FCGs of SLE patients. Graph metrics quantify various topological and functional characteristics of a given graph, resulting in a homogenous set of features. A further advantage of graph metrics is their capacity to summarize topological network features that may characterize underlying pathophysiology in a group of patients which would otherwise be obscured by individual differences in the connectivity indices between specific brain regions. Several studies with clinical populations have reported promising classification results when using graph metrics as the final features fed to an ML model [25,26]. In the present study, several complementary graph metrics were computed, namely global efficiency, local efficiency, node degree, betweenness centrality and eigenvector centrality. Selection of the most prominent graph metrics to serve as features in discriminating between NPSLE patients and healthy controls was achieved through a random forest classification algorithm [27], appropriate for the relatively high feature/subject ratio of the current data set. Finally, we employed a conservative method of cross-validation to optimize generalizability of results to real-world situations (nested cross validation). This method was adopted to address an often-overlooked problem in approaches that combine feature selection with cross-validation algorithms, namely potentially non-representative of real-world performance due to biased multiple feature sets.

The goal of the present work is to assess the accuracy and robustness of a data-driven approach to identify connectivity metrics that differentiate resting-state hemodynamic recordings obtained from lupus patients, manifesting neuropsychiatric symptoms, and recordings obtained from a gender and age-matched sample of healthy volunteers. It was hypothesized that by preserving individual differences in functional connectivity patterns and by employing graph metrics on the individualized sFCGs, our approach would be sensitive to functional brain disturbances that accompany perfusion impairments noted in previous studies [4,5].

## 2. Materials and Methods

### 2.1. Study Participants and Data Acquisition

#### 2.1.1. Participants

Our dataset consisted of 44 neuropsychiatric systemic lupus erythematosus (NPSLE) patients and 39 healthy control (HC) volunteers. NPSLE diagnosis was based on physician judgement, following a multidisciplinary approach and considering patient age and European League Against Rheumatism (EULAR) risk factors for NPSLE (antiphospholipid antibodies, prior neuropsychiatric manifestation, generalized disease activity, findings of conventional MRI imaging and other diagnostic procedures) [4]. Resting state fMRI data were recorded in the MRI Unit, University Hospital of Heraklion. The study was approved by the hospital review board, and the procedure was thoroughly explained to all participants, who signed informed consent before undergoing MRI. The demographic and clinical characteristics of all participants are presented in Table 1. 

#### 2.1.2. Impact of Disease Assessment

In addition to conventional clinical indexes of disease activity (Systemic Lupus Erythematosus Disease Activity Index (SLEDAI)) and accumulated organ damage (Systemic Lupus International Collaborating Clinics index (SDI)), a subgroup of patients (*n* = 35) were tested on two neuropsychological tests assessing visuomotor coordination efficiency (Trail Making Test Part A (TMT-A)), and visuomotor set shifting/mental flexibility (Trail Making Test Part B (TMT-B)). The former entails connecting encircled numbers, 1–25, placed pseudorandomly on an A4 sheet in ascending order [28], whereas TMT-B entails connecting encircled numbers and letters in an alternating and ascending fashion. Standardized time to complete each task adjusted for age and education level according to Greek population norms [29] served as the dependent variable. Correlational analyses between functional connectivity metrics that optimally differentiate NPSLE patients from healthy controls and these clinical/neurocognitive indices computed among NPSLE patients would help establish the functional significance of aberrant functional connectivity patterns in NPSLE. Self-reported symptoms of depression were recorded from 32 NPSLE patients using the Hospital Anxiety and Depression Scale (HADS) [30].

#### 2.1.3. MRI Acquisition

All brain MRI acquisitions were performed on a clinical, upgraded 1.5T whole-body superconducting imaging system (Vision/Sonata, Siemens/Erlangen), equipped with high performance gradients (Gradient strength: 40 mT/m, Slew rate: 200 mT/m/ms) and a two-element circularly polarized head array coil (minimum voxel dimensions: 70 μm × 70 μm × 300 μm). The basic imaging protocol consisted of a 3D T1-w MPRAGE (TR/TE: 1570/1.73 ms, 1 mm/1 NEX/160 axial sections), a T2wTSE (TR/TE: 5000/98 ms, 4 mm axial sections) and a Turbo FLAIR (TR/TE/TI: 9000/120/2320 ms, 4 mm axial sections) sequence. Axial sections were acquired parallel to the plane passing through the anterior and posterior commissures (AC–PC line). Conventional MR images were interpreted by a senior neuroradiologist (E.P.) with 20 years of experience, blinded to the clinical and laboratory data, who reported any incidental findings not related to SLE, or findings related to focal SLE morphological abnormalities, such as acute or old infarcts, hemorrhages and focal brain atrophy. Patients with any of the aforementioned MRI findings were excluded. Resting-state functional MRI (rs-fMRI) was derived from a T2*-weighted, fat-saturated 2D-FID-EPI sequence with repetition time (TR) 2320 ms, echo time (TE) 50 ms, field of view (FOV) 192 × 192 × 108 (x, y, z). Whole brain scans consisted of 36 transverse slices with 3.0-mm slice thickness and no interslice gap. Each BOLD time series consisted of 150 dynamic volumes (the first three volumes were discarded for the present analyses). Acquisition voxel size was 3 × 3 × 3 mm.

### 2.2. Data Preprocessing and Denoising

The fMRI images were smoothed, normalized and co-registered to the MNI space using SPM8. In terms of signal denoising, grey matter, white matter and cerebrospinal fluid (CSF) mean signals were regressed out of all voxel time series in order to mitigate their effects on fMRI BOLD time courses. The first five principal components of white matter and CSF regions were regressed out of the signal as well as their first order derivatives. These steps were completed using CompCor implemented within the CONN [31] preprocessing module and executed in MATLAB. The fMRI time series were detrended and bandpass filtered in the 0.008–0.09 Hz range in order to eliminate low frequency drift and high frequency noise. This frequency band is considered “standard” in many rs-fMRI studies [32], and it contains the low frequency fluctuations that are considered the most crucial part of the signal in rs-fMRI FC studies.

### 2.3. Functional Connectivity Graph Calculation and Statistical Filtering

The automated anatomical labeling atlas (AAL) [14] was used in order to parcellate the brain in 90 distinct spatial regions of interest (ROIs). Upon superimposing each region’s mask on the original image, voxel time series within each region were averaged, and one representative time series was obtained. Only these 90 representative ROI time series were utilized in the following analysis steps.

Pearson correlation coefficients were calculated for each pair of ROIs resulting in a functional connectivity graph (FCG) for each subject containing all the possible connection strengths between distinct cortical regions. The absolute connectivity values were retained, as the distinction between positive and negative correlations was not pertinent to this study and did not aid further analysis steps.

One thousand iterated amplitude adjusted Fourier transform surrogate time-series (IAAFT) were produced for each of the original subject’s 90 representative ROI time series. In the same manner as described above, an FCG was produced for each “set” or instance of the surrogate time series, thus resulting in one thousand surrogate connections for each pair of ROIs.

Next, one-sided *p*-values were obtained that corresponded to the likelihood that the observed correlation value could belong to the distribution of surrogate correlation values. This was calculated by estimating the proportion of surrogate connections that were greater than the “true” or actual connections [33]. The obtained *p*-values reflected the statistical significance of the respective ROI–ROI connection strength. FDR [34], with a false discovery rate of 0.05, was utilized in order to control for multiple comparisons. The connections found insignificant by this statistical filtering scheme were considered a product of spurious or random connection and therefore discarded.

It should be noted that tests were also conducted using a two-stage graph reduction scheme, initially filtering with surrogates and then with OMST (Orthogonal Minimum Spanning Trees) [20] as well with OMST as a standalone topological graph reduction technique. Better results in terms of model performance as well as the extent of graph reduction were achieved using the surrogate time series filtering method presented in this work.

### 2.4. Network Metric Calculation

Using the reduced FCG of each participant, we calculated the following graph measures: node degree, local efficiency, betweenness centrality and eigenvector centrality, computed for each node; global efficiency was also computed to characterize the entire FCG.

Local efficiency is closely related to the clustering coefficient, and it is analogous to the measure of global efficiency computed on the neighborhood of a given node. It can reveal whether a system is tolerant or immune to faults. In other words, it shows how efficient the communication is between the node in question and its immediate neighbors if it were removed from the graph. It is calculated as the inverse of the average of the shortest paths of a given node with all other nodes in the graph. The shortest path in a graph is defined as the path between two nodes that minimizes the sum of weights of its constituent edges. The characteristic path length or average shortest path is roughly the average distance between a pair of nodes in a graph. It is calculated as the average of shortest paths between all pairs of nodes/vertices in a graph.

Centrality in general characterizes the relative importance of a node/vertex in a network. Node degree can be considered a simple measure of centrality. Node degree of a graph is quantified by the number of vertices or links connected to a given node. Degree is a relatively simple measure, but it can reveal useful information about the immediate connectedness of each node; edge weights are not used in the calculation of degree.

More sophisticated measures of node centrality are betweenness and eigenvector centrality. Betweenness centrality (BC) can be used to estimate the amount of a given node’s influence on the overall flow of information in a graph. It can be used to identify a node that bridges two or more parts of a graph. Betweenness centrality of a node is calculated as the fraction of all shortest paths in a network containing that node. As a result, a high BC value would imply a node that participates in a large number of shortest paths.

Similarly, as a measure of centrality, eigenvector centrality (EC) is theoretically able to distinguish important hubs in a network, crucial to the overall connectedness of the graph. EC addresses the problem of quantifying centrality in a self-referential manner, meaning that for a node to have a high EC value, it must be connected to other nodes with high EC. A node’s EC value is derived as an eigenvector element, equivalent to the largest eigenvalue of the adjacency matrix.

Global efficiency is defined as the inverse of the average shortest path length. As the name implies, it is a global measure, referring to the communication efficiency of the entire graph.

For the calculation of betweenness centrality, and local and global efficiency, a simple transformation of the matrices is necessary, translating FCG values from weights to lengths, the simplest way being 1/FCG as the input matrix. This may be done internally in some cases by the chosen algorithm or package. Brain Connectivity Toolbox (BCT) [35] implemented in MATLAB was used for the calculation of all graph metrics. As suggested by BCT, betweenness centrality values were normalized by dividing them with (number of ROIs − 1) × (number of ROIs − 2). All the above network/graph metrics were combined in order to create the feature vector. The number of features in the initial feature vector were 361 (i.e., 4 metrics × 90 nodes plus a single global efficiency value) per subject.

### 2.5. Machine Learning

#### 2.5.1. Classification: Random Forest

The entire set of features were fed into a random forest (RF) classification model embedded in a nested cross validation (CV) scheme utilizing an RF feature importance based selection algorithm. Among available classification algorithms, RF [26] is noted for its simplicity, interpretability and ability to handle high dimensional data obtained from relatively small samples, demonstrating good generalizability. Additionally, the RF model handles the combination of different types of variables quite well (continuous or categorical) and has been successfully employed in several rs-fMRI FC studies [36,37]. RF belongs to the family of averaging, ensemble methods, based on randomized decision trees. It entails several “simpler” estimators or “weak learners”, which are built independently, and their predictions are averaged in order to produce the final prediction [26]. Averaging helps to increase predictive accuracy and control over-fitting. Randomness is introduced in the classifier construction process at two levels. Firstly, each learner uses a bootstrapped version of the training data; this is generally called bagging. The second level of randomness is introduced in the form of the randomly selected subset of variables split when a decision tree is growing. These two sources of randomness aim to decrease the variance of the final estimator, as individual decision trees typically exhibit high variance and a tendency to overfit. The main parameters of an RF classifier are the number of trees in the forest/ensemble, and the number of variables selected at each node.

A crucial function of the RF algorithm is to produce estimates of feature importance in order to evaluate individual feature contribution in the classification task and support feature selection—a necessary step in the present work given the large number of initial, computed features. Ideally, for the classification model to perform optimally, the initial number of features fed to the model should be substantially lower than the number of subjects in the data set [38]. The method used for estimating feature importance is mean decrease impurity (i.e., average decrease in node impurity over all trees for each individual feature).

Scikit-learn’s [39] implementation of the RF classifier in python was utilized in the classification and feature selection models. The parameters changed from their default values were those controlling the number of trees/estimators used, as well as the number of features that were considered when looking for the best split. These were changed according to the general instructions presented in the documentation and other similar studies, such as [37]. The values used were 300 trees/estimators in the forest, and all features were considered at each split. Higher numbers of trees were not deemed necessary and would also increase model runtime greatly with no significant benefit.

#### 2.5.2. Machine Learning Validation and Feature Selection

CV is typically employed in machine learning classification applications as an effective method of estimating the prediction accuracy and error of a given model. Besides providing vital information about a model’s real-world classification performance, CV is also an essential tool in the stage of model selection. The most often implemented CV methods in classification problems, including biomedical studies, are leave-one-out cross validation (LOOCV) [11] and k-fold cross-validation [7]. These methods have several drawbacks, especially when employed in combination with feature selection, the most crucial being that the classification results produced are possibly overoptimistic and not indicative of what can be expected in real world testing. Model overfitting is another potentially serious problem especially with relatively small sample sizes. Additional to these issues that can be avoided, the final models produced are often not generalizable, meaning the results are only valid for the dataset on which they were trained and do not indicate the model’s real-world performance with new data.

A proposed improvement to these CV methods is a variant of nested CV, which was chosen over LOOCV or k-fold CV in the present work for two main reasons: firstly, to ensure reliable identification of a minimum set of features that best characterize aberrant functional connectivity patterns in NPSLE; and secondly, to ensure high degree of generalizability in future studies through unbiased evaluation of the final model on previously untouched data.

**Figure 1:**
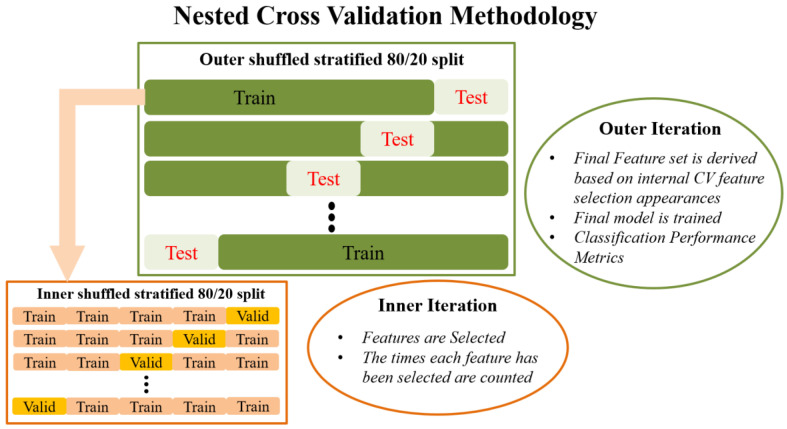
Graphical representation of the cross validation pipeline.

Externally, an 80/20 train–test split is applied to the data that randomly shuffles the data in each iteration. The internal CV operates only on the training portion of the data (80%). Feature selection in the traditional sense is performed in the internal iteration loop, on many different reshufflings of the 80% of the dataset (training subset of the external split). In each iteration of the internal CV, different combinations of the initial features are potentially selected by the RF algorithm, and features are ranked in order of importance. This information is saved across each iteration in the form of indices/names of the selected features. Next, the overall most frequently selected features throughout all internal iterations, i.e., appeared the most times, in the top ranked feature set are used in the external model. In this manner, the feature set on which the external model is tested is not derived from a single run (on a single random subset of data) of the feature selection algorithm, as is the case in most ML methodologies. Subsequently, the external classification model is trained with the entire 80% of the data, using the features selected as described above, and tested on the remaining 20% of the data that has been left completely untouched. The final prediction performance is indicated by the mean and standard deviation of the performance metrics obtained during the external testing. This is in turn repeated many times in order to eliminate the possibility of particularly favorable splits or cherry-picked evaluation subsets. Features used in the final classification model are also saved in order to report the overall most selected features across the external model iterations. The methodology analyzed above is visually depicted in Figure 1.

#### 2.5.3. Classification Performance Metrics

Next, a brief description of the classification performance metrics is given. Τhe accuracy score shows the overall accuracy of the model and is calculated as the ratio of the correctly classified samples by the total number of samples. Precision (or positive predictive value) reveals the ability of the classifier to not label a negative sample as positive, and it is calculated as the ratio of true positive samples by true positive plus false positive samples. Sensitivity/recall (true positive rate) shows the model’s ability to classify the patient (negative) cases correctly (as negative). It is calculated as 1 minus the false negative rate or as the ratio of true positives to true positives plus false negatives. The F1 score is essentially a combination of precision and recall, as its formula contains only these two metrics. As the harmonic average of two crucial model performance measures, F1 score is often used as a single metric in machine learning studies. The ROC (receiver operating characteristic) curve is computed by plotting the true positive rate against the false positive rate. Finally, computing the AUC (area under the curve) reveals the probability that the classifier will “favor” one of the two classes (HC or NPSLE). ROC-AUC is mostly used in the model comparison and selection stages of a study in order to ensure that the model does not classify (to an extreme extent) one of the classes with greater probability than the other. We estimated the required sample size needed to support the significance of an identified ROC curve compared to a null hypothesis value (0.5 or 50%) [40]. The probability of rejecting the null hypothesis (Type I error—alpha) was set to 0.05, Type II error (beta) was set to 0.10 corresponding to a power of 0.90 and the ratio of group sample sizes was 0.886. Sample size estimation calculations were performed using MedCalc for Windows, version 15.0 (MedCalc Software, Ostend, Belgium).

#### 2.5.4. Robustness of the Automated Preprocessing and Machine Learning Approach: AutoML

An important step towards the validation of the robustness of an automated preprocessing and machine learning pipeline is to produce stable findings across multiple executions. The pipeline proposed could potentially introduce bias due to the incorporation of statistical filtering of functional connections via surrogate analysis. Running the IAAFT algorithm, producing one thousand surrogates for every BOLD time series, twice, will create surrogates varying in amplitude. As a result, the distribution of connectivity strengths for every pair of ROI BOLD time series used as reference to retrieve statistically significant connections might differ. In order to control for these parameters, the entire pipeline was executed (and the results obtained) twice in order to compare the outcome in terms of performance metrics and the extracted important topological features.

### 2.6. Statistical Analyses

The clinical validity of the set of computed graph metrics following feature selection was assessed through Pearson correlation coefficients with TMT-A and TMT-B performance (time to complete each test). In addition, we compared the subgroup of patients displaying a higher degree of accumulated damage since the onset of the disease (as indexed by a score >0 on the SDI) with the remaining patients on each of the 12 graph metrics using one-way ANOVAs. All tests were evaluated at Bonferroni-adjusted a = 0.05/12 = 0.004.

## 3. Results

As shown in Table 2, optimal classification performance was characterized by balanced sensitivity (74.5%) and specificity (73.0%) in discriminating between NPSLE patients and healthy volunteers. These results were achieved using a nested cross validation scheme entailing an 80/20 train–test split, performed both internally and externally on the dataset, stratified using class labels in order to maintain equal ratios of the two groups. Performance reached a plateau after roughly 400 external and 40 internal iterations with negligible changes in performance at larger numbers of iterations (up to 1000 outer and 100 inner iterations were tested). The number of iterations, given the relatively limited size of the entire dataset, was considered able to examine a perfectly acceptable number of instances of train–test combinations for validation of the model to produce reliable results. The required sample size for supporting the area under the ROC curve compared to a random null classifier with a 0.5 or 50% by chance value was 57 subjects. Based on the adopted Type I and II error levels (0.05–0.10), our findings are significant with 30 positive cases (NPSLE subjects) and 27 negative cases (healthy controls) in a total sample size of 57 subjects compared to 83 subjects in both groups.

**Figure 2:**
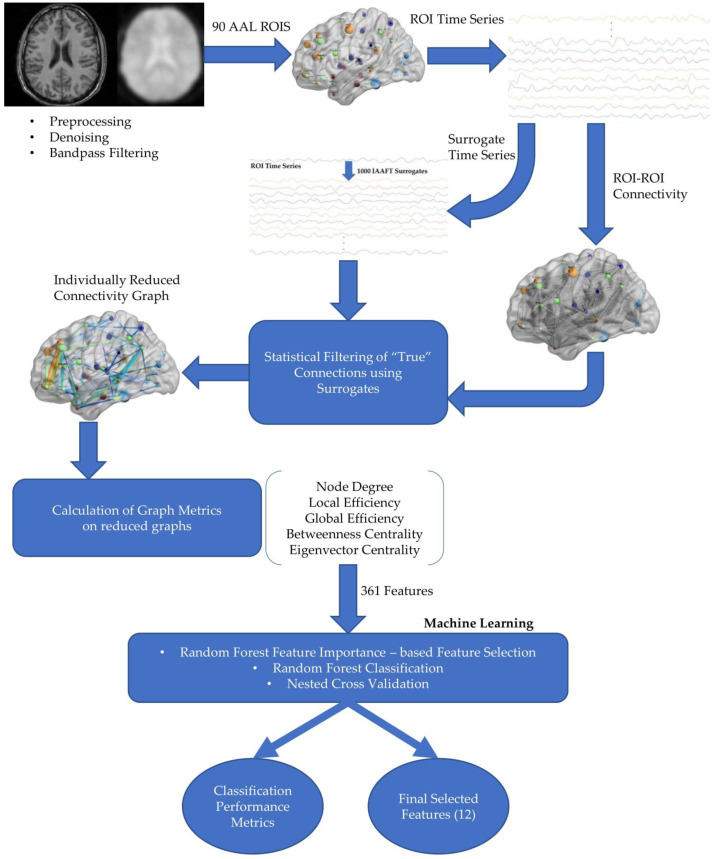
Diagram of proposed analysis pipeline.

Best classification tests were achieved with cross-validation models (internal and external) limited in the feature selection phase to 12 features, which are listed in Table 3 and illustrated anatomically in Figure 2 (created using BrainNet Viewer [41]). Inspection of average metric values revealed that the NPSLE group displayed higher graph metrics in the left angular gyrus (node degree and local efficiency), left anterior cingulate gyrus, right caudate, left inferior parietal lobule, and left superior temporal gyrus (betweenness centrality), right middle frontal gyrus (eigenvalue centrality), and right precentral gyrus (local efficiency). Conversely, higher values were noted among healthy participants in the left pars orbitalis and right parahippocampal gyrus (betweenness centrality) and the left superior frontal gyrus and left temporal pole (Eigenvalue centrality).

Although the model comprising 12 features outperformed all other models, classification accuracy for models retaining 9–18 features was only moderately inferior, while significant performance reduction was noted for models including fewer than 9 or more than 18 features. Considering the size of the dataset, this number of features is considered appropriate. Including a much greater number of features, problems related to the so called “curse of dimensionality” become pronounced (especially when the initial number of features is much greater than the number of subjects). On the other hand, using fewer features entails the risk of highly inflated performance due to random weak patterns present in one or two features alone, as well as overfitting the model and thus leading to a non-generalizable final model.

The proposed pipeline, including the preprocessing steps, construction of individualized whole-brain sFCG, extraction of important topological features and finally, its performance estimation in discriminating NPSLE from HCs was executed twice in its entirety. This was completed in order to ensure overall model robustness and stability. Quantitative classification performance metrics as well as the final selected features remained unchanged between tests.

In exploratory analyses, a higher local efficiency score in the left angular gyrus (*p* = 0.04) and a higher eigenvector centrality score in the temporal pole (*p* = 0.02) were associated with higher degree of accumulated damage, since the onset of the disease is indexed by a score > 0 on the SDI (although these differences did not reach significance). Among graph metrics that were found to be higher among NPSLE patients as compared to healthy controls, local efficiency in the angular gyrus was associated with worse performance on TMT-B (r = −0.48, *p* = 0.003), and betweenness centrality in the caudate was associated with worse performance on TMT-A (r = −0.53, *p* = 0.001). Conversely, higher local efficiency in the precentral gyrus was associated with better performance on TMT-A (r = 0.57, *p* < 0.001).

Finally, to assess whether individual differences among NPSLE patients (and potentially the observed differences from controls) could be attributed to comorbid depression symptoms, we computed Pearson correlations between each of the 12 network metrics and HADS depression score. Results failed to identify substantial associations in the NPSLE group (r < |0.2|, *p* > 0.2).

## 4. Discussion

In the present work, we designed a pipeline suitable for deriving sparser static functional connectivity graphs from rs-fMRI time series without a-priori assumptions regarding prominent patterns of connectivity. The proposed algorithm was applied to the full connectivity graph from each participant consisting of 90 sections of neocortex, basal ganglia, cerebellum, thalamus and medial temporal regions (as specified in a standard anatomical map). Initially, connection weights were statistically thresholded using surrogate data to ensure that each connectivity weight was evaluated for significance by considering person- and region-specific dynamic characteristics of the recorded time series. In this manner individual differences in the precise outline of thresholded FCGs were preserved and summarized by a set of graph metrics. Next, feature selection was conducted using a technique that is not prone to overfitting (random forests), while the classification performance of selected metrics was evaluated using a conservative nested cross validation scheme.

Feature selection and classification results revealed that optimal differentiation between the two groups could be achieved based on 12 graph network metrics involving 11 ROIs. The metrics specified in the final model (node degree, betweenness/eigenvector centrality, local efficiency) may be considered as positive indices of the degree of importance of a particular node (ROI) in the sparse network of brain regions characteristic of each participant. Several ROIs identified in the best-performing model closely correspond to the regions identified in two recent studies as displaying hypoperfusion in NPSLE as compared to healthy controls, namely the superior frontal gyrus [4,5] and the superior parietal gyrus [4]; and the temporal pole, cingulate gyrus, precentral gyrus, middle frontal gyrus and inferior frontal gyrus [5]. Moreover, the present results complement the work of [6], who reported increased connectivity strength both within and between standard resting-state FC networks [13] by highlighting the role of specific brain regions for global connectedness.

There are, therefore, indications of uncoupling between functional connectivity and regional perfusion; we found evidence that certain regions previously noted for reduced perfusion may have a more prominent role within the functional connectivity network in NPSLE patients as compared to healthy controls (i.e., superior parietal gyrus, precentral gyrus, orbital middle frontal gyrus and cingulate gyrus). In the case of the right precentral gyrus, its apparently enhanced role within the functional network of brain areas appears to serve a compensatory function, in view of the positive association of local efficiency and visuomotor performance. Further, the cingulate gyrus is part of the limbic circuit; evidence of enhanced engagement of this region (indexed by higher BC in the NPSLE as compared to the HC group) may relate to the higher frequency of potentially significant depressive and anxious symptomatology among NPSLE patients noted in the present sample, in accordance with the clinical literature [42,43]. This hypothesis, however, was not corroborated by correlational analyses between BC values (or graph metrics in the remaining 10 ROIs for that matter) and self-reported depression symptoms among NPSLE patients.

Finally, the increased role within the functional connectivity network of NPSLE patients was found for three areas not previously noted to show perfusion disturbances (caudate nucleus, left inferior parietal lobule and adjacent angular gyrus). Associations with neurocognitive measures suggested that the apparent enhancement in functional connectivity in the caudate and angular gyrus was detrimental for visuomotor performance and mental flexibility, respectively.

### 4.1. Topologically Filtering of the Fully-Weighted sFCG with Surrogates

An important step before estimation of functional connectivity graphs is the adaptation of a functional atlas that divides the human brain into regions of interest. Then, we obtained a BOLD time series from each of these regions of interest to characterize the hemodynamic activity of each brain area. In our study, we adopted the famous AAL atlas with a total of 90 brain areas (45 per hemisphere). From these 90 BOLD time series, we estimated functional connectivity strength in every pair of time series employing Pearson’s correlation coefficients, resulting in 4005 independent pairwise functional connectivity values. The majority of functional neuroimaging studies either used the full-weighted functional connectivity graph for identifying disease-specific patterns of connectivity disturbances (e.g., [44,45]), or focused on a-priori defined networks involving relatively small sets of brain areas (e.g., [6]). In both studies as well as others, an assumption was made that the large set of pairwise functional connectivity strengths exists or is significant compared to a reference. Due to lack of an individual reference, we borrowed techniques designed by physicists, namely surrogate data analysis [15,16,17]. To address this problem, we constructed one thousand surrogate time series per ROI BOLD time series using the iterated amplitude adjusted Fourier transform surrogate time-series (IAAFT) [18]. IAAFT corrected the flatness bias problem observed in the power spectra of conventional AAFT time series. This statistical filtering approach revealed person-specific, non-random network topology without relying on group averaging.

### 4.2. Feature Selection and Classification Algorithm

In contrast to several other methodologies, the combination of feature selection, classification and nested CV scheme used in the present study helped to identify a limited set of connectivity features ensuring moderate diagnostic accuracy. This is ensured by including in the final subset of the feature vector only the features which were beneficial in highlighting crucial patterns present in a large portion of the data. This method provides a more robust approach for extracting potential biomarkers related to the disease and its diagnosis. It should be noted than in preliminary analyses, tests were also conducted using a two-stage graph reduction scheme, initially filtering with surrogates and then with OMST [46] and with OMST as a standalone topological graph reduction technique. Better results in terms of model performance as well as the extent of graph reduction were achieved using the surrogate time series filtering method presented in this work.

Results from the various tests performed with a wide range of feature set sizes as well as train–test set sizes are suggestive of adequate model stability to ensure generalization of results. These changes include the number of inner and outer iterations, changes in the two model parameters mentioned (number of trees and features considered at splits), as well as the number of features selected in each iteration. The specific features derived from the final model, across all outer iterations, remained relatively the same with changes mostly limited to the ranking position in terms of overall importance. Additionally, the entire pipeline was run twice to assess possible bias in the surrogate generation process. This implies quite strongly that the set of topological brain connectivity measures presented here could prove effective in the detection of NPSLE in a similar experimental setup. This conclusion is supported by (a) significant convergence between the final set of discriminating features identified in the present study and regions identified as hypoperfused in previous studies with NPSLE patients, using diverse methodologies, and (b) significant associations between several of these connectivity metrics and disease severity or neurocognitive capacity.

While building upon an earlier report [12], where a robust machine learning model was developed for the classification of NPSLE patients and HCs using prespecified resting-state functional connectivity networks, two new goals were set. The first improvement was to tackle this diagnostic and exploratory problem using a fully data-driven method. This was of paramount importance given the manual selection of networks and regions performed in the previous study as well as in several others on similar datasets and diseases. This goal was met, as all cortical regions were included using the AAL atlas and the reduction of FCGs on a subject level was performed in a data-driven fashion, comparing the connectivity strengths to the distribution of those obtained using 1000 surrogate time series. Graph metrics offered an elegant solution as they allow the comparison of the greatly different functional connectivity networks (due to subject-level FCG reduction) by deriving several topological network measures.

In addition, graph metrics can provide useful insights into important and differentiating network characteristics. With the exception of global efficiency, all graph metrics utilized in the present study are calculated per node, i.e., per ROI. Thus, the final derived features represent indices of the relative importance of a given ROI. In this manner, brain regions of great importance within the global functional connectivity networks characterizing each group can be the subject of further investigation. The second, more indirect goal of the study, was to identify a parsimonious set of connectivity features which could be independently validated in future samples as biomarkers for the diagnosis of NPSLE. This is in reference to a relatively small set of regions most significantly affected (or not affected) by the disease as well as more complex network characteristics of the diseased brain’s connectedness.

A final note concerns the metrics used for model evaluation—primarily recall and F1 score (as a harmonic average of precision and recall). Given the potential clinical implications of classification results, reducing the possibility of false negative predictions is of paramount importance. The main classification performance metric indicating such behavior is recall (sensitivity = 1 − false negative rate). Recall scores as well as those of the other performance metrics are considered perfectly acceptable considering the more conservative performance estimation afforded by nested CV. Avoiding model overfitting and using a conservative method for estimating classification performance are the two factors contributing to the results being indicative of real-world performance with new subjects.

**Figure 3:**
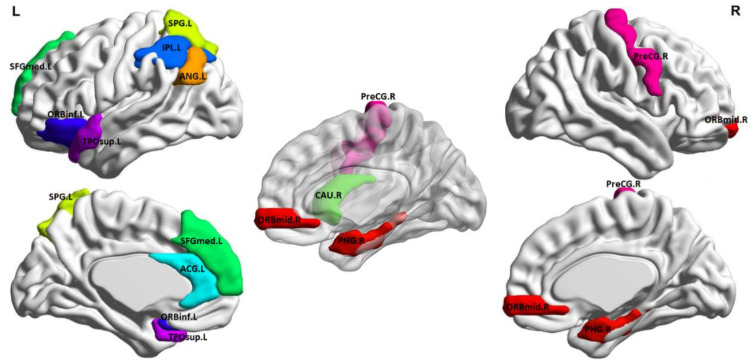
Outline of the 11 ROIs as specified in the automated anatomical labeling atlas (AAL) map, which provided the 12 graph metrics in the best-performing classification model discriminating between NPSLE patients and healthy controls. ROI abbreviations are explained in Table 3.

In sum, automated machine learning (AutoML) is a rapidly growing field mainly involving computer scientists with a medical background working as a bridge between clinicians and computer scientists with expertise in software engineering and data analysis able to harness machine and deep learning tools [47]. We hope that our study will be helpful to non-experts to learn about good practices in fMRI analysis and the construction of robust connectomic biomarkers by adopting suitable machine learning algorithms. Compared to similar attempts to develop “automated” disease diagnosis tools such as in the present [8,9,11], the level of complexity of the proposed methodology is comparable. The steps of the entirely data-driven analysis and classification pipeline (as shown in Figure 3) are not few but are characterized by relative simplicity, interpretability and independence from the designer with minimal tuning.

## 5. Conclusions

### Limitations—Future Work

One of the limitations of this study concerns the relatively high standard deviation of the classification performance metrics, especially specificity. Increasing the number of subjects, should they become available, would probably help reduce the standard deviation of these results and provide further assurance of model robustness. Furthermore, the CV methodology would benefit greatly from a larger number of subjects, given that in nested CV, the data is being split twice, internally and externally, reducing the number of subjects in the internal train and test subsets.

In order to extend the work presented here and test whether the conservative and unbiased model developed stands up to the design’s expectations, testing on a new external dataset of NPSLE patients and healthy controls should be performed. Firstly, the proposed model could be used in its final form, with the measures derived as significant on the present dataset. Testing could be conducted on a new, independent validation cohort to test model real-world generalization ability. Additionally, the regions and network characteristics implied by the final network measures should be studied further as biomarkers of NPSLE by taking into account individual variability in clinical patient characteristics (such as disease duration, types of neuropsychiatric manifestations, etc.) to better understand their significance for the pathophysiology of the disease. Future work could focus on potentially systematic patterns of temporal variability functional network connectivity that may characterize aberrant brain function in NPSLE [48]. Finally, methods borrowed from biology and genetics–genomics could be evaluated for their ability to provide helpful results [49].

## Figures and Tables

**Table 1:** Demographic and clinical characteristics of NPSLE patients and controls.

	Healthy Participants (*n* = 39)	NPSLE Patients (*n* = 44)	*p*-Value
Female	34 (87.2%)	43 (95.6%)	0.07
Age, mean (SD in years)	42.9 (15.4)	44.2 (12.7)	0.7
Disease duration, mean (SD in years)	--	6.3 (6.1)	--
SLEDAI, mean (SD)	--	4.3 (2.8)	--
SDI score, mean (SD)	--	0.32 (0.56)	--

NPSLE: neuropsychiatric systemic lupus erythematosus (SLE); SLEDAI: Systemic Lupus Erythematosus Disease Activity Index; SDI: Systemic Lupus International Collaborating Clinics index.

**Table 2:** Mean and standard deviation of classification metrics.

Metric	Mean ± Standard Deviation
Accuracy (%)	73.5 ± 10
Precision (%)	76.5 ± 12
Sensitivity (Recall) (%)	74.5 ± 15.5
Specificity (%)	73 ± 16.5
F1 Score (%)	74.5 ± 11
ROC-AUC (%)	73.5 ± 10.5

**Table 3:** Most significant features associated with the best-performing classification model differentiating NPSLE patients from healthy controls (HC).

Metric	x, y, z Coordinates in MNI Space	ROI	Hemi-Sphere	Region Code in AAL Map
		NPSLE < HC		
BC	−35.98 30.71 −12.11	Inferior frontal gyrus, pars orbitalis	L	ORBinf.L
BC	25.38 −15.1 −20.47	Parahippocampal gyrus	R	PHG.R
EC	−4.8 49.17 30.89	Superior frontal gyrus, medial	L	SFGmed.L
EC	−39.88 15.14 −20.18	Temporal pole	L	TPOsup.L
		NPSLE > HC		
ND	−44.14 −60.82 35.59	Angular gyrus	L	ANG.L
LE	»	»	L	»
BC	−4.04 35.4 13.95	Anterior cingulate gyrus	L	ACG.L
BC	14.84 12.07 9.42	Caudate nucleus	R	CAU.R
BC	−42.8 −45.82 46.74	Inferior parietal lobule *	L	IPL.L
EC	33.18 52.59 −10.73	Middle frontal gyrus, orbital part	R	ORBmid.R
LE	41.37 −8.21 52.09	Precental gyrus	R	PreCG.R
BC	−23.45 −59.56 58.96	Superior parietal gyrus	L	SPG.L

Abbreviations; EC: eigenvector centrality, BC: betweenness centrality, LE: local efficiency, ND: node degree; L/R: left/right. * Excluding, supramarginal and angular gyri.
